# Morphometric analysis of patient-specific 3D-printed acetabular cups: a comparative study of commercially available implants from 6 manufacturers

**DOI:** 10.1186/s41205-022-00160-w

**Published:** 2022-11-07

**Authors:** Harry Hothi, Johann Henckel, Sean Bergiers, Anna Di Laura, Klaus Schlueter-Brust, Alister Hart

**Affiliations:** 1 The Royal National Orthopaedic Hospital, Stanmore, HA74LP UK; 2 The Institute of Orthopaedics and Musculoskeletal Science, University College London, London, UK; 3 Department of Orthopaedic Surgery, St. Franziskus Hospital Köln, 50825 Cologne, Germany

**Keywords:** 3D printing, Micro-CT, Porosity, Additive manufacturing

## Abstract

**Background:**

3D printed patient-specific titanium acetabular cups are used to treat patients with massive acetabular defects. These have highly porous surfaces, with the design intent of enhancing bony fixation. Our aim was to characterise these porous structures in commercially available designs.

**Methods:**

We obtained 12 final-production, patient-specific 3D printed acetabular cups that had been produced by 6 manufacturers. High resolution micro-CT imaging was used to characterise morphometric features of their porous structures: (1) strut thickness, 2) the depth of the porous layer, (3) pore size and (4) the level of porosity. Additionally, we computed the surface area of each component to quantify how much titanium may be in contact with patient tissue. Statistical comparisons were made between the designs.

**Results:**

We found a variability between designs in relation to the thickness of the struts (0.28 to 0.65 mm), how deep the porous layers are (0.57 to 11.51 mm), the pore size (0.74 to 1.87 mm) and the level of porosity (34 to 85%). One manufacturer printed structures with different porosities between the body and flange; another manufacturer had two differing porous regions within the body of the cups. The cups had a median (range) surface area of 756.5 mm^2^ (348 – 1724).

**Conclusions:**

There is a wide variability between manufacturers in the porous titanium structures they 3D print. We do not currently know whether there is an optimal porosity and how this variability will impact clinically on the integrity of bony fixation; this will become clearer as post market surveillance data is generated.

## Background

Additive manufacturing (AM), commonly referred to as 3D-printing, is revolutionising orthopaedic implant engineering. This technology is used in numerous off-the-shelf implant designs but has demonstrated its greatest clinical impact by enabling patient-specific titanium cups to be printed for the treatment of patients with large and complex shaped acetabular defects [1, 2]; without which many of these patients would otherwise have been unable to be reconstructed and walk.

3D printing also enables manufacturers to print highly porous surfaces with the design intent of enhancing short- and long-term bony fixation [3]. There are currently no agreed standards in place to guide how this porosity should be designed and consequently manufacturers have different approaches to this. It is important that these differences are understood to inform the post-market surveillance of these devices and to aid understanding of which porous strategies achieve the optimal fixation with bone.

High resolution micro–Computed Tomography (micro-CT) has previously been used to characterise these porous structures in conventionally sized, off-the-shelf AM acetabular shells [4–6]. This has demonstrated wide variability between manufacturers in the morphometric features of their porous designs. A similar investigation of patient-specific acetabular implants has not previously been performed.

In this study we obtained 12 final-production, patient-specific 3D printed acetabular cups that had been produced by six manufacturers and used micro-CT imaging to characterise and compare their porous structures.

## Methods

### Materials

**Fig. 1:**
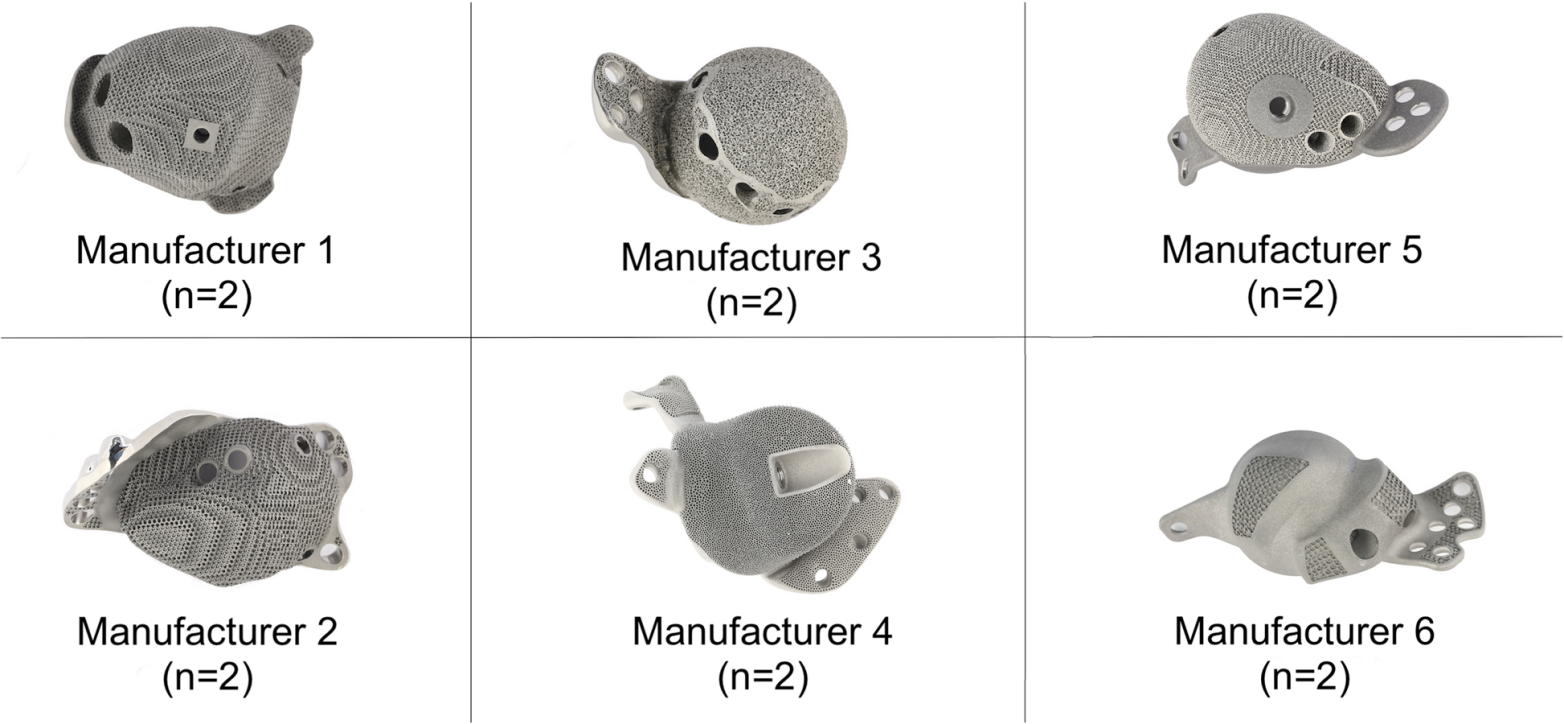
3D printed patient-specific acetabular cups produced by 6 manufacturers were examined in this study, consisting of 2 designs from each manufacturer (12 cups total)

This study examined 12 final-production, unused titanium alloy acetabular cups that had been 3D printed to a customised shape and size for the treatment of patients with large acetabular defects. These consisted of two patient-specific implant designs manufactured by each of the six leading manufacturers of 3D printed orthopaedic implants, Fig. 1. All designs consisted of the main acetabular body and flanges for fixation. In all cases, the implants had exceeded their 6-month window in which they could be used in patients after the date on which they had been designed.

We randomly assigned each manufacturer with an identifier of between Cup_1 and Cup_6; the two cups from each manufacturer were randomly assigned a label of A or B. From this point on, the implants will be referred to as Cup_1A, Cup_1B, Cup_2A, Cup_2B etc. when referring to each of the two implants from the six manufacturers.

### Micro-CT imaging

High resolution 3D imaging of each cup was performed using a Nikon XTH 225 micro-CT scanner (Nikon Metrology, Tring, United Kingdom). The cups were mounted as close as possible to the beam source whilst still capturing the entire component in the field of view and scanning was performed with a beam current and voltage of up to 150 μA and 225 kV respectively. Scans were captured in increments of 0.11° for a total of 3177 frames set to an exposure of 1000 ms. A 1 mm thick copper filter was fitted in front of the beam source in order to minimise any beam hardening induced when scanning a metal sample.

### Reconstruction of micro-CT data

The captured two-dimensional (2D) projection images were first reconstructed in CT Pro 3D software (Nikon Metrology, UK), utilising a filtered back-projection algorithm. Second-order polynomial-correction numerical filtering was incorporated into the algorithm to further minimise any beam hardening that may have occurred. The spatial resolution of the reconstructed data was 45 μm.

### Morphometric analysis of micro-CT data

**Fig. 2:**
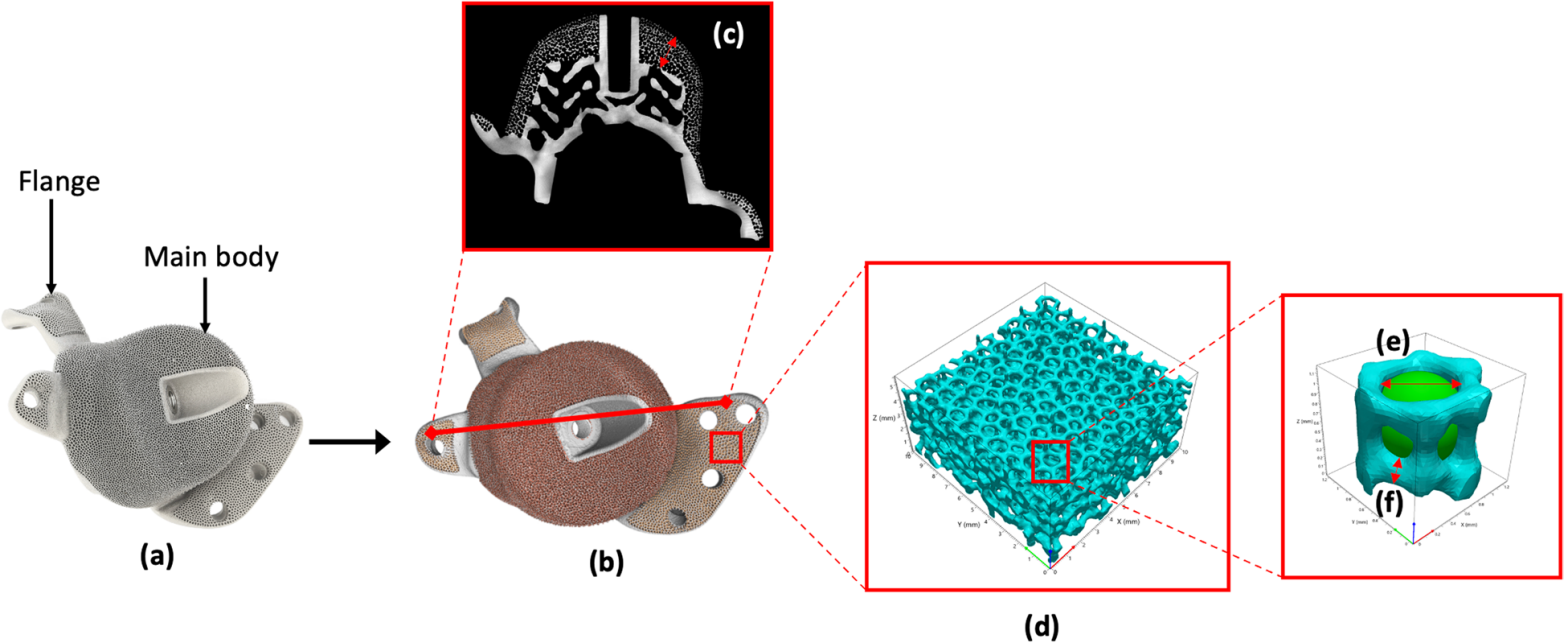
Summary of the analysis performed during which (**a**) the unused patient-specific 3D-printed implants were obtained, (**b**) the implants were micro-CT imaged, reconstructed for computational analysis and the variation in porosity within a single cup mapped, (**c**) the 2D scan slices were used to determine the maximum and minimum depth of the porous structure, (**d**) samples from the porous regions were extracted to determine the level of porosity, (**e**) sub-samples were used to determine pore diameter and (**f**) analysis of the mesh struts was used to determine the wall thickness of the porous structure

The filtered and correct micro-CT data was then imported into the analysis software packages Volume Graphics (Heidelberg, Germany) and Simpleware (Synopsis, Exeter, UK) in order to measure the following parameters within the porous structures of each implant, Fig. 2:

1. the wall thickness of struts
2. the depth of the porous layer
3. the pore size
4. the level of porosity

Measurements of these four parameters were captured from a flange of each cup and from the main body. Additionally, we computed the total surface of each component.

### Strut thickness measurement

The reconstructed data was first imported into the analysis software Volume Graphics. The ISO-50 threshold was applied to segment the implant from the background and 3D volume renders of each component were subsequently generated. The wall thickness analyses function within Volume Graphics was implemented to the external porous layers of the implant in order to measure the thickness of the struts within the porous mesh. This analysis was then repeated in an inverted manner so as to determine the size of the voids within the mesh. Combining these analysis methods enabled a colour map of the variability of the porous structure on a single cup to be evaluated; this was used to determine if the manufacturer had printed the main cup body and flanges with different porosities and/or if the main body had a uniform porosity.

### Porous layer depth measurement

We imported the 2D projection images into Simpleware in order to characterise the depth of the porous layer. This was achieved through examination of the 2D images and a determination of the minimum and maximum depths.

### Pore size measurement

A multi-Otsu thresholding algorithm was applied in Simpleware to the 2D images in order to separate the titanium implant from the background (air) and render a segmented 3D model of each cup.

Three 10 × 10x10mm samples of the porous structures within the main cup body were captured computationally for analysis. In cases in which the depth of the porous region was less than 10 mm, we captured the maximum depth possible in each sample. Our previous analysis within Volume Graphics revealed that both implants from one of the manufacturers had two differing porous regions within the main body; we captured three samples from both of these regions.

Additionally, 5 × 5x5mm samples were computationally taken from the porous structures of a flange of each cup. The flanges of both cups from one manufacturer did not have a porous layer and so no samples could be captured from these flanges. In cases in which the porous layer on the flange was less than 5 mm deep, we captured the maximum depth that was possible.

The pore size of the mesh structure of each sample was measured by best fitting a sphere to the internal surface of a single mesh unit. This was performed on three unique units within each sample.

### Porosity measurement

The 10 × 10x10mm and 5 × 5x5mm samples previously captured from the main body and flange respectively, were used to determine the level of their porosity, expressed as a percentage. The volume of a cuboid completely enclosed within each sample was compared to the volume of the mesh material within this space using the equation below.

$$Porosity\,\left(\%\right)=\frac{Total\,Cuboid\,Volume-Mesh\,Volume}{Total\,Cuboid\,Volume}\times 100$$

### Surface area measurement

The surface area of each implant was calculated using a Simpleware function to assess the 3D model view data, in preference to a voxel measurement. The surface area of each face that formed the 3D model was calculated and summed.

### Statistical analysis

We performed Mann–Whitney U tests to determine if there were any significant differences in the morphometric parameters investigated between the cup body and flange of each cup (where applicable) and between different porous regions of the cup body (where applicable). All analysis was performed using the statistical software package Prism (GraphPad, La Jolla, California) and throughout, a *p*-value < 0.05 was considered statistically significant.

## Results

**Fig. 3:**
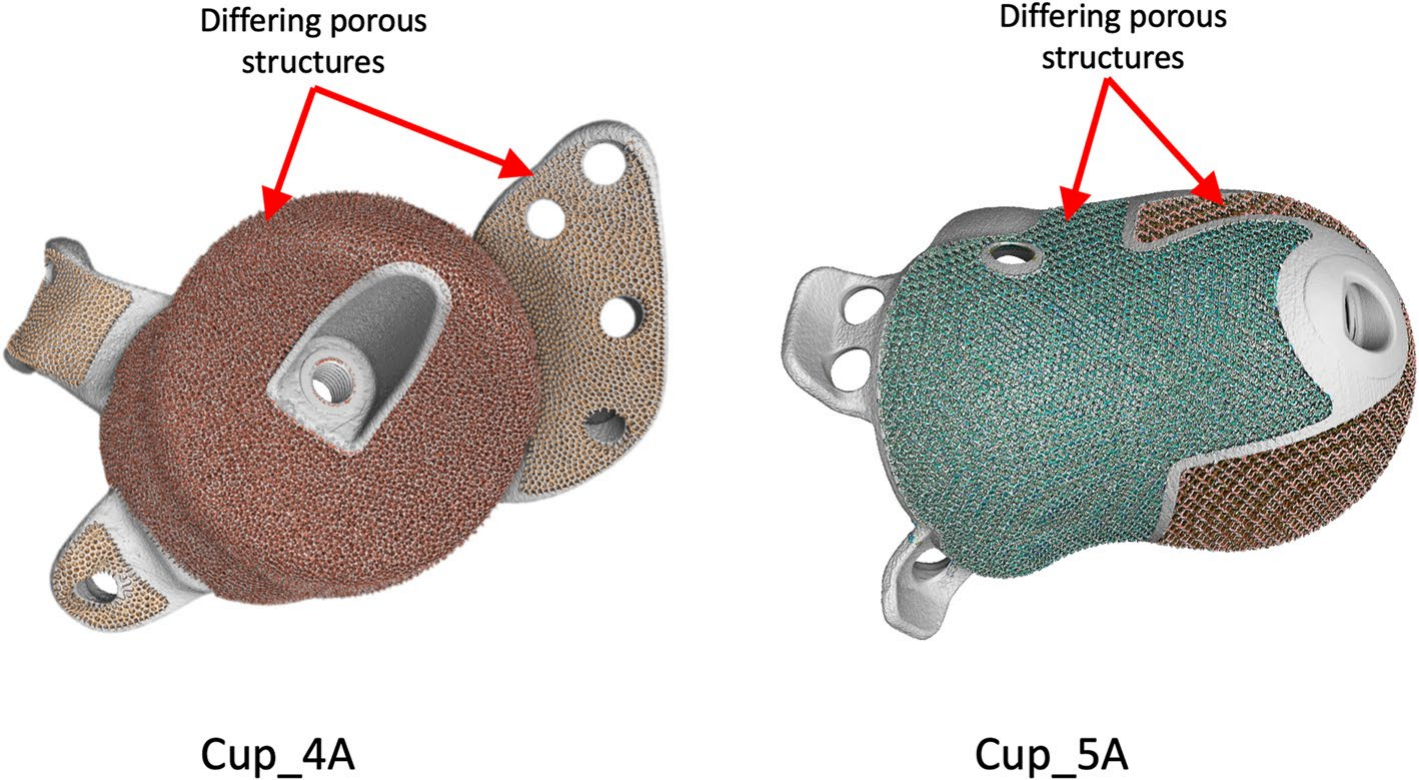
Colour mapping of the porous regions on the cups revealed a variability in Cups 4 and 5. The examples presented here show in Cup_4A, a difference between the porosity of the body and the flange, and in Cup_5A, two distinct porous regions within the cup body

All cups had porous layers on their main bodies. Cups 1–4 and 6 had porous layers on their flanges whilst Cup_5A and 5B did not. Colour mapping of the porosity on each cup revealed that Cups 1–3 and 6 had a uniform level of porosity across their body and flange. Both Cup_4A and 4B were found to have been printed with a different porosity between body and flange, whilst both Cup_5A and 5B had two distinct levels of porosity on their main bodies, Fig. 3.

Table 1 summarises the data captured for the five parameters investigated across all cups in this study.

**Table 1:** Summarising the morphometric parameters that were defined for each cup

	**Median Pore Size**, *mm* (IQR)	**Median Strut Thickness**, *mm* (IQR)	**Porous Layer Thickness**, *mm*		**Porosity, *%***	**Surface Area,** *mm*^*2*^
			**Minimum**	**Maximum**		
**Cup_1A**	1.37 (1.35 – 1.40)	0.62 (0.49 – 0.92)	0.71	4.14	57.15 (56.59 – 57.97)	1030
**Cup_1A Flange**	1.37 (1.34 – 1.42)	0.54 (0.41 – 0.82)	1.38	2.23	57.65 (51.71 – 60.73)	
**Cup_1B**	1.33 (1.29 – 1.40)	0.60 (0.47 – 0.53)	0.77	4.01	51.90 (51.28 – 54.95)	685
**Cup_1B Flange**	1.36 (1.30 – 1.41)	0.57 (0.43 – 0.80)	0.99	3.21	57.32 (52.60 – 58.75)	
**Cup_2A**	1.67 (1.66 – 1.69)	0.47 (0.40 – 0.57)	0.68	11.51	68.85 (68.21 – 69.42)	739
**Cup_2A Flange**	1.66 (1.63 – 1.72)	0.45 (0.36 – 0.55)	0.86	1.36	62.11 (57.20 – 70.83)	
**Cup_2B**	1.54 (1.52 – 1.54)	0.65 (0.56 – 1.06)	0.57	11.04	52.87 (47.68 – 53.28)	1724
**Cup_2B Flange**	1.54 (1.54 – 1.57)	0.59 (0.43 – 0.83)	0.73	1.20	56.53 (54.94 – 59.31)	
**Cup_3A**	1.24 (1.12 – 1.36)	0.28 (0.21 – 0.39)	0.86	2.21	75.58 (67.48 – 80.42)	399
**Cup_3A Flange**	1.09 (0.83 – 1.21)	0.29 (0.22 – 0.39)	0.57	1.16	71.88 (70.56 – 74.61)	
**Cup_3B**	1.50 (1.24 – 1.73)	0.28 (0.21 – 0.39)	1.00	1.17	69.77 (67.62 – 75.50)	348
**Cup_3B Flange**	1.27 (1.11 – 1.41)	0.31 (0.23 – 0.42)	1.14	1.44	71.32 (69.94 – 73.63)	
**Cup_4A**	0.96 (0.91 – 0.98)	0.38 (0.30 – 0.53)	0.70	7.26	66.66 (66.04 – 67.00)	1117
**Cup_4A Flange**	0.78 (0.77 – 0.81)	0.56 (0.43 – 0.95)	1.71	2.84	39.20 (37.62 – 40.17)	
**Cup_4B**	0.94 (0.91 – 0.97)	0.41 (0.33 – 0.59)	1.22	6.91	63.92 (62.95 – 64.99)	1127
**Cup_4B Flange**	0.74 (0.72 – 0.76)	0.63 (0.47 – 1.09)	1.23	3.34	34.32 (31.98 – 36.76)	
**Cup_5A_1**	1.87 (1.85 – 1.90)	0.45 (0.38 – 0.54)	4.94	6.20	85.02 (84.90 – 85.49)	774
**Cup_5A_2**	1.23 (1.16 – 1.26)	0.47 (0.33 – 0.63)	0.8	1.36	68.33 (65.92 – 72.33)	
**Cup_5B_1**	1.83 (1.79 – 1.89)	0.48 (0.41 – 0.60)	5.28	6.54	84.34 (83.90 – 84.42)	372
**Cup_5B_2**	1.13 (1.10 – 1.19)	0.51 (0.38 – 0.66)	0.93	1.67	67.84 (66.44 – 71.08)	
**Cup_6A**	1.63 (1.26 – 1.78)	0.45 (0.01 – 3.47)	0.98	1.08	80.29 (74.10 – 81.91)	217
**Cup_6A Flange**	1.34 (1.14 – 1.55)	0.49 (0.01 – 3.47)	0.99	1.14	77.88 (74.90 – 81.54)	
**Cup_6B**	1.89 (1.81 – 2.02)	0.53 (0.01 – 5.62)	0.96	1.30	73.91 (69.87 – 75.02)	321
**Cup_6B Flange**	1.62 (1.32 – 2.09)	0.53 (0.01 – 4.15)	0.96	1.26	73.65 (71.24 – 79.13)	

### Strut thickness measurement

**Fig. 4:**
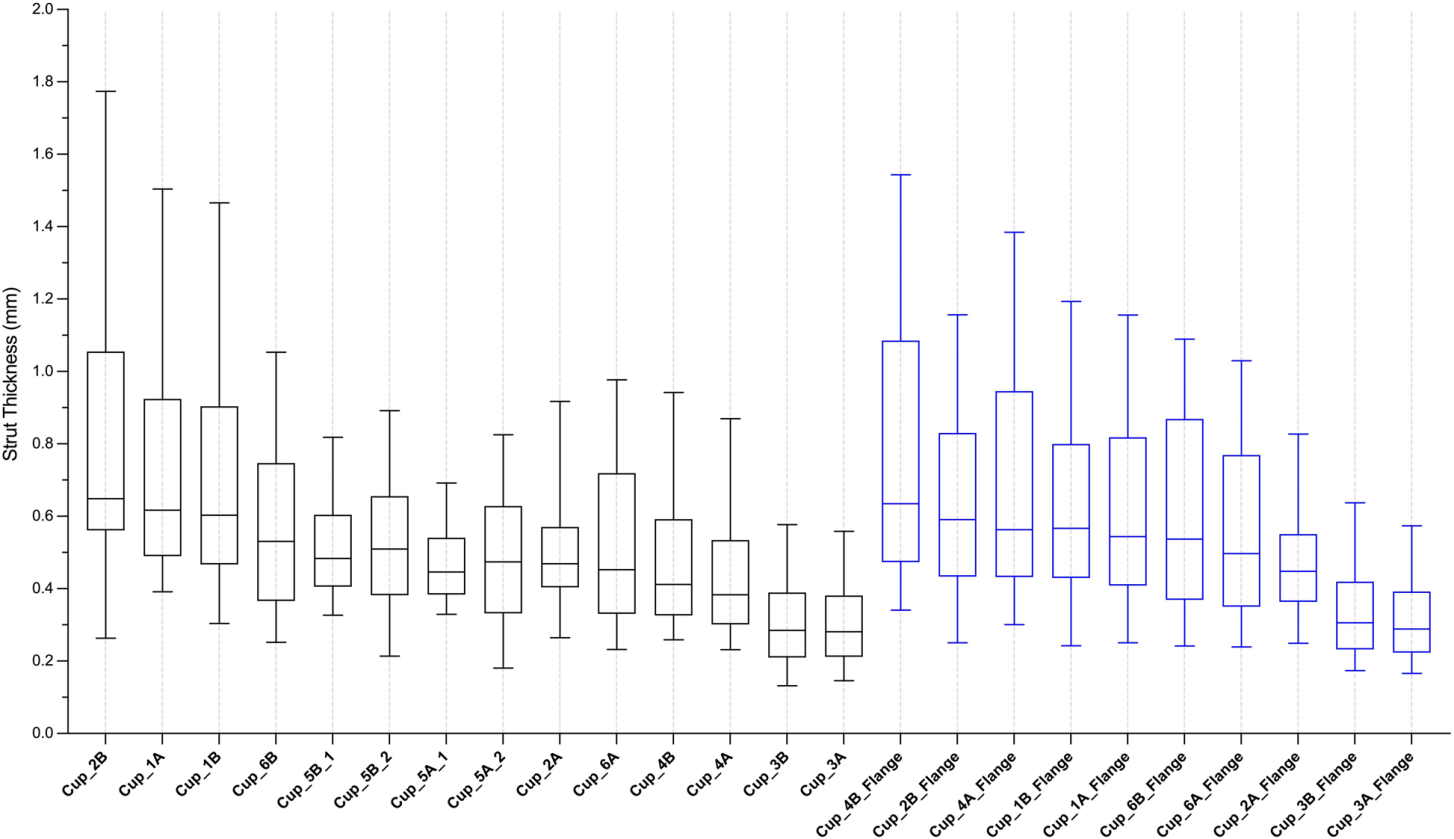
Box plots of the strut thicknesses measured from samples extracted from the main body backside and flange of each cup. The wall thicknesses are presented in descending order

The measures of the thicknesses of the struts within each cup are presented in Fig. 4. The median thicknesses of struts within the cup bodies ranged between 0.28 and 0.65 mm; this ranged between 0.29 and 0.63 mm within all flanges.

Cups 4A and 4B had thicker struts at their flanges compared to their bodies (0.56 mm & 0.38 mm, and 0.63 mm & 0.41 mm respectively). The strut thicknesses were comparable between body and flange for Cups 1–3 and 6. There was no difference in the strut thickness between the two porous regions of both Cup_5A and Cup_5B.

### Porous layer depth measurement

**Fig. 5:**
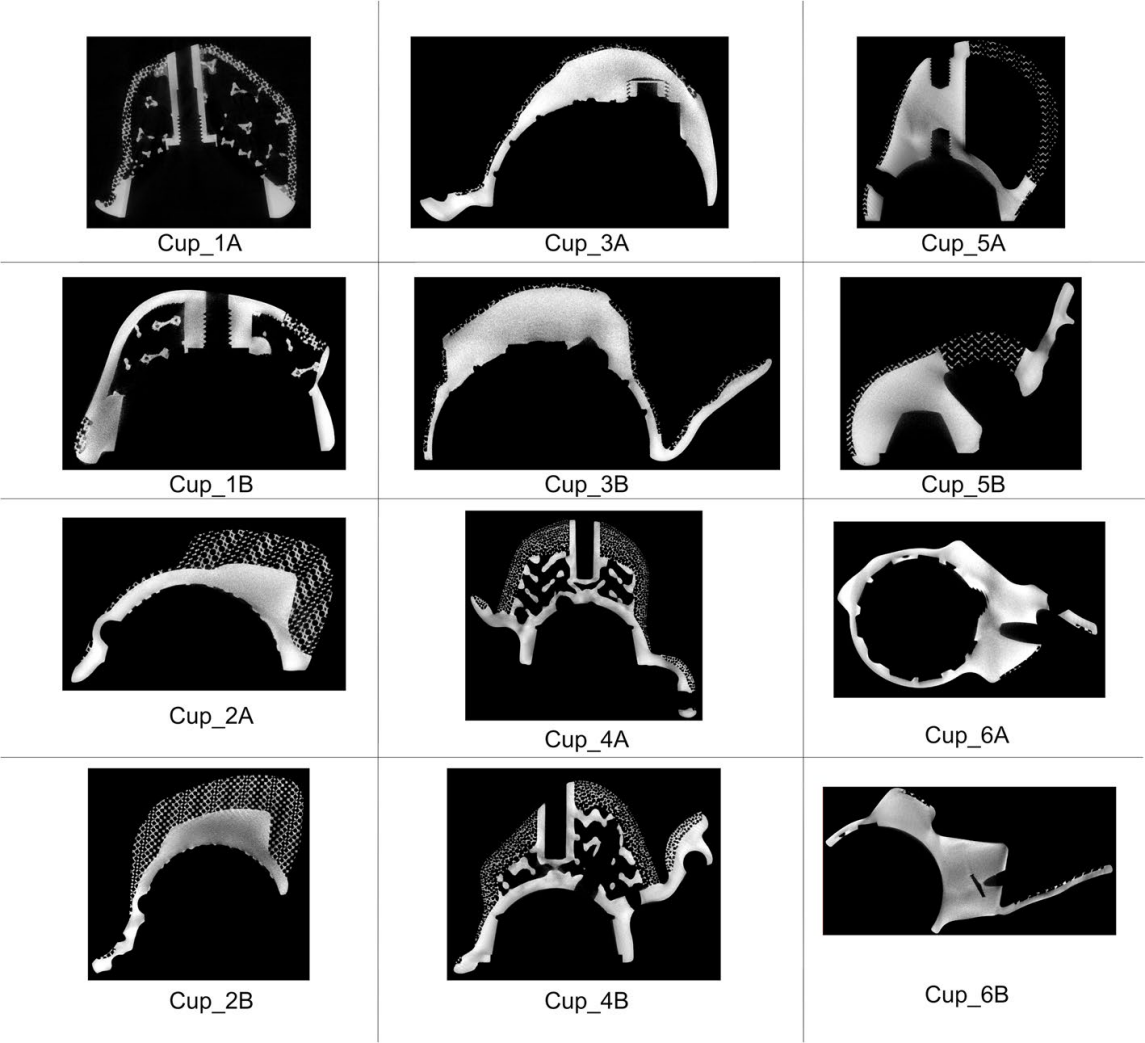
Examples of the 2D scan images that were examined for each cup in order to identify the minimum and maximum depths of the porous structures. These illustrate the variability of the porous depths on the body and flange of the different designs. The examples presented were selected to try and include regions in which the shallowest and deepest porous were present

**Fig. 6:**
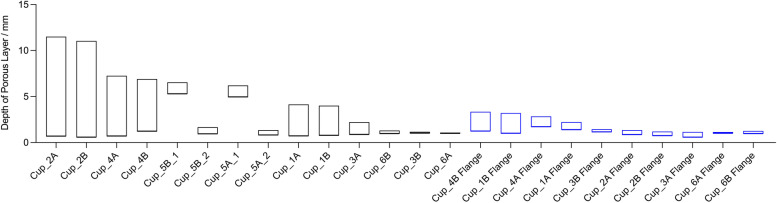
Box plots of the minimum and maximum porous depths measured for each cup; measurements were captured separately for the body and flange

Figure 5 presents examples of cross-Sect. 2D micro-CT scan images that were used to evaluate the depth of the porous layers. The minimum and maximum measures of depth are summarised in Table 1 and presented as box plots in Fig. 6. The porous depths ranged between 0.57 and 11.51 mm across all cup bodies and between 0.57 and 3.34 mm across the flanges. With the exception of Cup_3B, the range of depth measures for the flanges fell within the ranges for their corresponding measures on the main body. The maximum depth of the porous layer on Cup_3A was greater than in Cup_3B (2.21 mm and 1.17 mm respectively). There was a clear difference in the depths between the two porous regions on the bodies of both Cup_5A (6.20 mm and 1.36 mm) and 5B (6.54 mm and 1.67 mm).

There was a wide variability in the maximum depths across the designs, with depth in Cup_2A (for example) measured as almost tenfold greater than in Cup_3B.

### Pore size measurement

**Fig. 7:**
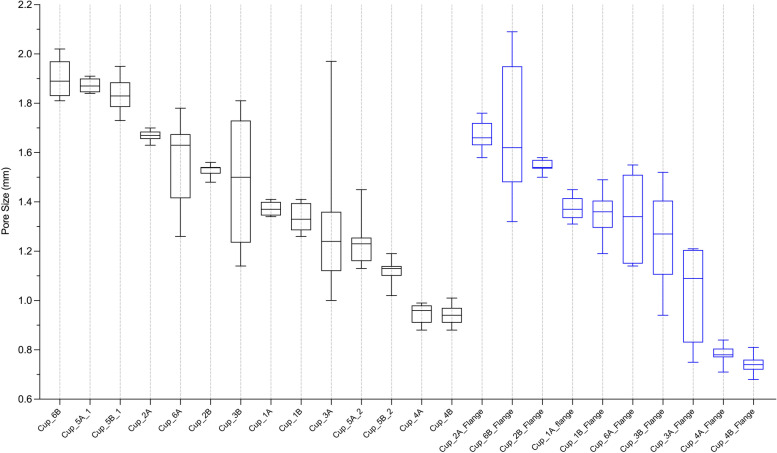
Boxes plots of the pore diameter measured from samples extracted from the main body and flange of each cup. The data is presented in order of decreasing diameter

Figure 7 presents box plots of the pore sizes of each of the cup bodies and flanges analysed.

The median pore size within the cup bodies ranged between 0.94 and 1.89 mm; this ranged between 0.74 and 1.62 mm within all flanges.

Comparison of the pore sizes between the body and flange of individual cups showed no difference for Cups 1 and 2. Cups 3 and 6 appeared to have larger pores within their main bodies however the range of sizes measures for this design of implant were significantly greater than in the other designs, due to the irregular nature of the porous design. Cups 4A and 4B had pore sizes that were significantly greater at their bodies by a median of 0.18 mm and 0.20 mm respectively, *p* < 0.05. Both Cups 5A and 5B had significantly different pore sizes between the two porous regions within their main body, by 0.64 mm and 0.70 mm respectively, *p* < 0.05.

### Porosity measurement

**Fig. 8:**
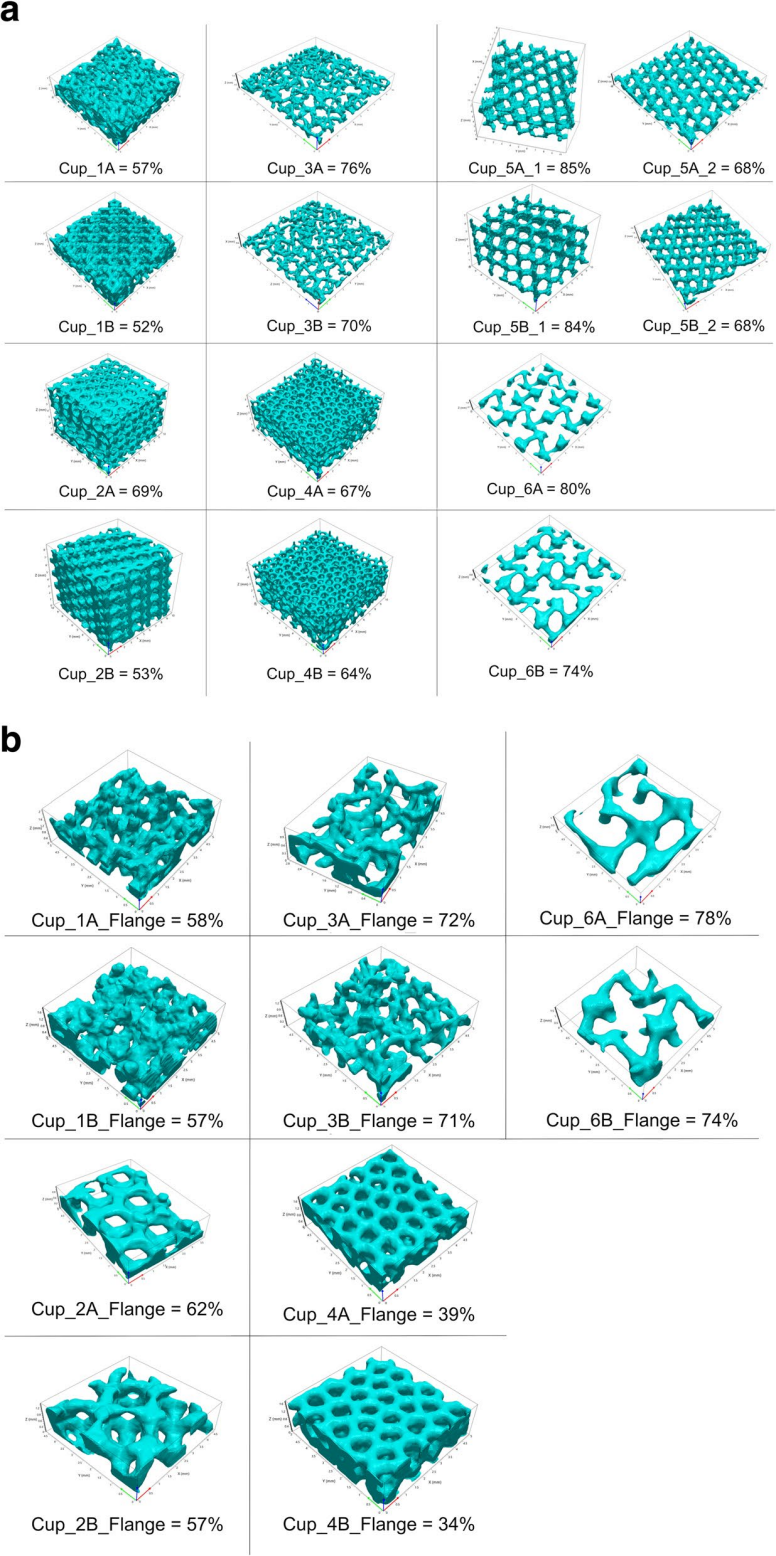
**a** Presenting examples of the samples that were extracted computationally from each cup body, together with the median porosity value that was calculated. Both Cups 5A and 5B had two differing porous regions. **b** Presenting examples of the samples that were extracted computationally from a flange on each cup, together with the median porosity value that was calculated. Both Cups 5A and 5B did not have a porous layer on their flanges

Figures 8a and 8b present examples of the samples that were computationally extracted from the porous regions of the body and flange, together with the median porosity that was measured for each cup. The porous regions of Cups 1, 2, 4, 5 and 6 were observed as having engineered lattice structures based on a design of triply periodic minimal surfaces (TPMS); Cups 3A and 3B appeared to have a stochastic lattice structure within their porous regions. The porosity within the cup body ranged between 52 and 85%, whilst for the flanges, porosity was measured to be between 34 and 78%. The porosity of the body and flange of Cup_4A were 67 and 39% respectively; this difference was significant (*p* < 0.01). Cup_4B also had significantly different porosities between body and flange; 64 and 34% respectively, *p* < 0.01. Cups 1, 2, 3 and 6 had comparable measures of porosity between body and flange.

The porosity of the two cup body regions of Cup_5A were significantly different; 68 and 85%, *p* = 0.0079. Cup_5B also had significantly different porosities on its body; 67 and 84%, *p* = 0.0079.

### Surface area measurement

The median (range) surface area of the 12 cups was computed as 756.5 mm^2^ (217 – 1724).

## Discussion

This study is the first to present an independent comparative analysis of final-production, commercially available, patient-specific 3D printed implants. We found that a high variability exists between manufacturers in the design of their bone-facing porous structures. This variability is seen in the thickness of struts, how deep the porous layers are (0.57 mm—11.51 mm), the pore size, the level of porosity (34—85%) and the uniformity of this across the body and flanges of the cups. This data will allow surgeons, researchers, regulators and manufacturers to better understand the relationship between the design of 3D printed implants and their future clinical performance.

We do not know which design offers the optimal solution for achieving long-term fixation with bone, or indeed if we will observe any clinical differences at all; this understanding will come mainly from long-term follow up studies and registry data. An important factor influencing the performance of these types of patient-specific implants is the severity of the defect being treated and the quality of the underlying bone [2, 7]. The broad variability in the structure of this bone may in part explain the variability in porous designs seen between manufacturers, particularly as the evidence of long-term performance is still being generated. Trabecular bone is known to have a porosity of between 50 and 90% and pore sizes in the ranging from as low as 50 to 300 μm to as much as 1 mm [8–10].

The two parameters impacting the degree of porosity within a given volume are the strut thickness and the pore size; decreasing the strut thickness and/or increasing the pore size will generate a more porous volume. In the current study we found that variability in porosities between manufacturers was primarily due to the variability of the pore sizes, which had a difference of 1.13 mm between the greatest and lowest median measures of each cup. The difference in the greatest and lowest median strut thickness was 0.37 mm. 3D printing offers manufacturers the advantage of being able to control the pore sizes of their lattices with greater ease. This is particularly important as we still do not know the optimal pore size that should be used in these implants. Some previous experimental studies have suggested that pores between 100 and 500 μm are required for optimal bone ingrowth to occur [11–13], whilst another study suggests a minimum of 150 μm is required [14], which contrasts with other studies indicating that pore sizes should be between 300 and 1,000 μm for optimal bone ingrowth [15]. Conversely, a different study has suggested that pore sizes of greater than 1,000 μm may be optimal to maximise bone growth [16].

The implants examined in the current study had median pore sizes of between 740 and up to 1,870 μm, indicating a design trend towards printing pores that are larger in size. It is not clear if this trend is influenced by a difficulty in reliably printing smaller sizes.

Interestingly, there was a 20-fold difference between the shallowest and deepest porous surfaces measured in this series of implants, ranging from 0.57 mm to as deep as 11.51 mm. Previous experimental studies have suggested that bone ingrowth can occur up to a maximum of approximately 2.5 mm within porous structures [17]. The ‘minimum’ depth of bone growth necessary for stable mechanical fixation to occur is not clearly defined, and conversely the clinical advantage of porous surfaces deeper than 2.5 mm is not clear in respect to bone growth. Deeper porous layers may however other advantages such as reducing the mass of the implant and altering its mechanical properties at the implant-bone interface to enable some level of micro-motion to occur, which is known to encourage bone growth [18].

We found almost an eightfold difference in the smallest and largest surface area measured for the cups. These differences are unsurprising and are likely a reflection of the variability in the anatomies of the patients the implants were designed for. Notably, the surface area of these patient-specific 3D printed implants is considerably greater than the surface area of conventional shells, which we’ve measured at our centre as being in the order of 311 cm^2^ (compared with a median of 756.5 cm^2^ in the current study). This greater surface area is due in part to the dimensionally larger size of the implants but also due to the porous lattice structures that have been printed, meaning that more titanium surface is exposed to patient tissue than in conventional implants. Whilst titanium is known to be highly biocompatible [19], it seems prudent that this potential increase in the exposure to titanium is monitored. We recommend that clinical studies investigate the utility of blood testing of titanium levels in these patient groups as a means of monitoring function. This testing may also help understanding of if any residual titanium powder from the printing process is released whilst in situ.

Future studies investigating these types of implants should seek to understand the impact of any differences in post-processing used between manufacturers. This may include a characterisation of partially melted titanium powder beads that have previously shown to remain present on implants, even after they have undergone post-processing [4]. The analysis of these implants should also extend in the future to investigating the presence of any structural defects that may exist within the printed structures, as has shown to occur in some off-the-shelf cups [20].

## Conclusions

This study is the first to present comparative analysis data of final-production, commercially available patient-specific 3D printed acetabular implants. Our micro-CT imaging of 12 cups printed by 6 manufacturers showed a wide variability in their porous structures, in relation to: (1) strut thickness, (2) the depth of the porous layers, (3) pore size and (4) the level and uniformity of porosity across the implants. We do not know how this variability in the porous structures will impact clinically on the integrity of bony fixation; this will become more clear as long-term post-market surveillance data is generated.

## Data Availability

The datasets used and/or analysed during the current study are available from the corresponding author on reasonable request.
